# Graph mining for next generation sequencing: leveraging the assembly graph for biological insights

**DOI:** 10.1186/s12864-016-2678-2

**Published:** 2016-05-06

**Authors:** Julia Warnke-Sommer, Hesham Ali

**Affiliations:** College of Information Science and Technology, University of Nebraska Omaha, Omaha, NE 68182 USA; Department of Pathology and Microbiology, University of Nebraska Medical Center, Omaha, NE 68198 USA

**Keywords:** Next generation sequence assembly, Graph mining, Metagenomics, Crohn’s disease

## Abstract

**Background:**

The assembly of Next Generation Sequencing (NGS) reads remains a challenging task. This is especially true for the assembly of metagenomics data that originate from environmental samples potentially containing hundreds to thousands of unique species. The principle objective of current assembly tools is to assemble NGS reads into contiguous stretches of sequence called contigs while maximizing for both accuracy and contig length. The end goal of this process is to produce longer contigs with the major focus being on assembly only. Sequence read assembly is an aggregative process, during which read overlap relationship information is lost as reads are merged into longer sequences or contigs. The assembly graph is information rich and capable of capturing the genomic architecture of an input read data set. We have developed a novel hybrid graph in which nodes represent sequence regions at different levels of granularity. This model, utilized in the assembly and analysis pipeline Focus, presents a concise yet feature rich view of a given input data set, allowing for the extraction of biologically relevant graph structures for graph mining purposes.

**Results:**

Focus was used to create hybrid graphs to model metagenomics data sets obtained from the gut microbiomes of five individuals with Crohn’s disease and eight healthy individuals. Repetitive and mobile genetic elements are found to be associated with hybrid graph structure. Using graph mining techniques, a comparative study of the Crohn’s disease and healthy data sets was conducted with focus on antibiotics resistance genes associated with transposase genes. Results demonstrated significant differences in the phylogenetic distribution of categories of antibiotics resistance genes in the healthy and diseased patients. Focus was also evaluated as a pure assembly tool and produced excellent results when compared against the Meta-velvet, Omega, and UD-IDBA assemblers.

**Conclusions:**

Mining the hybrid graph can reveal biological phenomena captured by its structure. We demonstrate the advantages of considering assembly graphs as data-mining support in addition to their role as frameworks for assembly.

**Electronic supplementary material:**

The online version of this article (doi:10.1186/s12864-016-2678-2) contains supplementary material, which is available to authorized users.

## Background

Next Generation Sequencing (NGS) technologies have made it possible to directly sequence environmental samples to detect and analyze the components of biological communities. As a growing number of exciting discoveries are being made in this field of metagenomics, it is becoming increasingly clear that we are intricately connected to and influenced by the host of microorganisms known as the human microbiome. The importance of the human microbiome has been recognized, so much so that it has been referred to as the forgotten organ of the human body [1]. The commensal and pathogenic microorganisms populating the human body have been found to play major roles in metabolism [2, 3], immune system maturation and modulation [4, 5], and even in the development of various types of cancers [6, 7].

In metagenomics studies, NGS machines produce short DNA sequences called reads which are randomly sampled at a very high coverage from environmental DNA. These reads are extremely short in comparison to the bulk DNA amount in environmental samples. Illumina technologies currently are capable of producing NGS reads anywhere from 125 bps to 300 bps in length with output capabilities ranging from 25 million to 3 billion reads per run [8]. Reads produced by the 454 technologies are up to 1000 bps in length with data set sizes reaching 1 million reads [9]. More recently introduced technologies such as PacBio are able to produce reads at longer read lengths exceeding 10 k bps in length [10].

The short length of current NGS reads makes it difficult to extract any useful information from any read individually. Therefore, multiple analytical approaches have been developed to organize, aggregate, and analyze short read sequences. The high coverage of next generation sequencing technologies means that the reads in a data set will be sampled from a biological sample such that many of them overlap. These overlap relationships can be used to order the reads into a representation of the original sequence region. For the purpose of facilitating downstream analysis, many applications require that read sets are assembled into longer stretches of sequence called contigs. Assembly tools often rely on the mathematical structure called a graph to organize and model the short sequencing reads. Two graph theoretic approaches are typically followed in assembly, overlap graph based approaches and de Bruijn graph based approaches [11]. In the overlap graph based approaches, each read is mapped to a unique node in the overlap graph. If two reads overlap, then their corresponding nodes will be connected by an edge. An ordering of the reads is found by traversing the nodes in the overlap graph. The second graph-based approach relies on the de Bruijn graph as its graph theoretical foundation. In this approach each read is broken into all possible k-mers. The k-mers become edges in the de Bruijn graph. For each k-mer, its left and right k-1-mers become nodes in the de Bruijn graph. In this approach, a read ordering is found by traversing the edges in the de Bruijn graph.

Numerous different assembly tools have expanded on these two graph theoretic foundations introduced in the previous paragraph. The assemblers IDBA [12] and SPAdes [13] build and integrate de Bruijn graphs for multiple values of *k*. When *k* is too small this often results in many branches in the de Bruijn graph; however, when it is too large this results in gaps in the de Bruijn graph [12]. These iterative de Bruijn graph approaches mitigate this problem by taking advantage of all values of *k*, resulting in longer produced contigs [12, 13]. Similarly, other assemblers such as SGA [14] have made use of the string graph, which simplifies the overlap graph by eliminating redundant edges [15, 16].

Assemblers optimized for single genome assembly are unlikely able to handle the complexities of metagenomics data sets. Metagenomics-specific assembly tools have been developed to address some of the challenges of metagenomics assembly including the presence of conserved and repetitive sequence regions, which introduce branching paths and tangles within the assembly graph. Assembly tools typically extend contigs along a maximal non-branching path in the assembly graph. A branch point in a path forces an assembler to either terminate contig extension or to select a branch with which to continue extension - the branch selected may or may not be correct, introducing error into the assembled contig. The metagenomics assemblers Omega and UD-IDBA analyze the read and kmer coverage differences between paths that compose branch points in the assembly graph for the purpose of resolving them [17, 18]. The assembler MAP integrates mate-pair information into the assembly graph to resolve these branch points [19]. Machine learning has been used by the assembler MetaVelvet-SL to distinguish chimeric nodes from non-chimeric nodes [20].

The assembly of short reads is an aggregative process during which the global and local read relationship and therefore global and local genome architecture information is lost as reads are merged into flat contigs. In contrast, assembly graphs are information rich models that can capture features of the global architecture of the input genomic sequence [16] and have been mentioned in passing to be capable of capturing biological features such as conserved regions, rRNA operons, and horizontally transferred sequences [21]. However, there have been a very limited number of studies demonstrating the assembly graph’s power as an information rich data-mining support especially in metagenomics. Instead, the primary goal of most assembly tools is to improve the assembly process to produce longer and more complete assemblies. In this research, an expanded assembly graph, which is called the hybrid graph, is shown to be an excellent data-mining support that can be used to extract structural signatures associated with biological features and make novel biological discoveries.

We have developed a novel hybrid graph model that represents different regions of sequence data at different levels of granularity [22]. This hybrid graph model forms the foundation of the assembly and analysis pipeline called Focus. The model is constructed by creating a set of graphs produced by successive graph coarsening initialized on the original overlap graph. Nodes are integrated from different levels of the graph set into a hybrid graph to create a concise yet feature rich view of the input data set. Repeats and conserved intergenomic regions are reduced within the hybrid graph, while global architecture is preserved. Local read overlap relationships are maintained in earlier levels of the coarsened graph set.

The Focus algorithm was applied for a study on read data sets obtained from the gut microbiomes of healthy individuals and individuals with Crohn’s disease. The overarching goal of this research was to explore the distribution of transposase genes and associated antibiotic resistance genes across bacterial genera in the gut microbiomes of healthy individuals and individuals with Crohn’s disease. The approach and results in this manuscript might provide insights into candidate genera for which horizontal gene transfer of transposon sequences and associated antibiotic resistance genes has occurred. We divide our study into three specific aims.

1. *Demonstrate that repetitive and transposable elements are associated with node characteristics*. To facilitate efficient extraction of meaningful graph structures in this study, each node in the hybrid graph is assigned a Shannon’s index score to reflect the local diversity of the various species or sequence regions in which the sequence represented by the given node is conserved or repeated. The Shannon’s index captures the number of edges incident to a given node as well as their evenness or how equally their weights are distributed. In the hybrid graph, each node represents a contiguous sequence region. Edges represent overlap relationships that this sequence region has with other contiguous sequence regions. An edge between two given nodes is weighted according to the summation of the read overlap lengths between reads composing the first sequence region with the reads composing the other sequence region. If a sequence region is repeated multiple times in a single genome or is present in multiple species, it might follow that its representative node in the assembly graph will have multiple in and out edges representing different sequence regions or species. In contrast, a node that is part of a single path in the assembly graph might be representative of a unique genomic region.
  Bacterial transposons are mobile DNA segments that can independently replicate and insert themselves within the same chromosome or plasmid or into a different chromosome or plasmid [23]. They have been implicated in the horizontal transfer of genes between different bacterial species. Transposase and integrase sequences are often a part of transposable elements and are commonly involved in their transfer. The simplest of bacterial transposons is the insertion sequence (IS) element shown in Fig. 1a, which is composed of two inverted repeats flanking genes necessary for transposition. The rDNA operon is a prevalent large repeat class in microbial genomes, ranging from 1–15 copies per genome [24]. In this manuscript, it is shown that nodes assigned with transposase/integrase genes and rRNA operon DNA had a greater proportion of high Shannon’s index scores in comparison to nodes assigned with other gene categories from the SEED subsystems (q = 2.44 × 10^−04^; paired Wilcoxon tests).

**Fig. 1:**
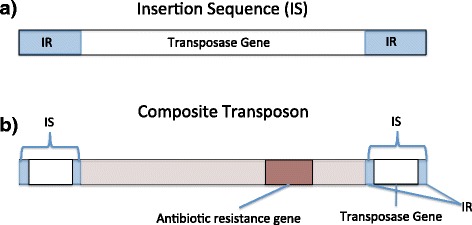
**a** The insertion sequence (IS) is the smallest transposon present in bacterial chromosomes and plasmids. It is composed of two inverted repeats flanking genes necessary for transposition. **b** The composite transposon is composed of two IS elements flanking a central protein coding DNA region. This central region often contains genes for antibiotics resistance
2. *Identify and characterize the phylogenetic distribution of antibiotic resistance gene classes associated with transposase/integrase sequences in healthy individuals and individuals with Crohn’s disease.* The human microbiome has been described as a reservoir for antibiotic resistance genes [25, 26] and as a hot spot for horizontal gene transfer [27] between bacterial taxa. Antibiotic resistance genes are often found in bacterial composite transposons, which are composed of two IS elements flanking a protein coding sequence region as shown in Fig. 1b [28], allowing their rapid spread between bacterial groups. Crohn’s disease is a chronic disorder where the gastrointestinal tract is inflamed [29]. Horizontal gene transfer has been suggested to be increased between pathogenic and commensal bacteria in inflamed gastrointestinal systems [30]. Furthermore, this population is more likely to be treated with antibiotic regimens for secondary complications such as bacterial overgrowth and abscesses [31]. Antibiotic use has been shown to increase antibiotic resistance in those to whom it is prescribed [32]. Due to these issues, it is important to understand the antibiotic resistance genes present in populations affected by Crohn’s disease.
  In a novel graph-mining approach, the structure of the hybrid graph is used to identify transposase/integrases sequences that might be located in multiple sequence regions (i.e. repeated in the same genome or distributed across multiple species) according to their assigned Shannon’s index score. Local graph exploration of the neighborhood surrounding these transposase and integrase sequences reveal associated antibiotic resistance genes. Clustering transposase sequences based upon their phylogenetic distribution obtained from the hybrid graph revealed several differences between the Crohn’s disease and healthy data set. Most transposase genes in the healthy data sets were clustered into a large *Bacteroides* group significantly enriched for tetracycline, macrolide-lincosamide-streptogramin B, and beta-lactamase antibiotic resistance genes. Transposase genes in the Crohn’s disease data sets were more diverse across phylogenetic groups including an *Enterococcus* cluster significantly enriched for aminoglycoside, macrolide, and streptogramin antibiotics resistance genes. This approach reveals clusters of genera for which transposase associated antibiotic classes are enriched and may provide insight into candidate bacterial groups in which horizontal gene transfer has occurred.
3. *Perform a competitive assembly evaluation of the assembler against other well-known assembly tools.* In addition to being a data-mining support, Focus is a strong assembly algorithm. In this study, a subset of the metagenomics data sets is assembled with Focus in a comparative study against the Omega [17], IDBA-UD [18], and MetaVelvet [21] assemblers. These assemblers were chosen for the comparison because they were metagenomics-specific assemblers. Furthermore, two of the assemblers, IDBA-UD and MetaVelvet, were based on the de Bruijn graph approach for assembly. One of these assemblers, IDBA-UD, is based on an iterative de Bruijn graph approach. The final assembler, Omega, is an overlap graph based assembler. These assemblers represent a wide range of graph-based approaches to which we compared Focus.
  Results demonstrate the knowledge that can be obtained from structural features of the assembly graph. Nodes annotated with several genetic features that are distributed across multiple species or are often present in multiple copies (rRNA) have a significantly greater proportion of high Shannon’s index scores than other nodes in the hybrid graph. This reflects a greater number of unique sequences that overlap with the genomic regions of these particular nodes. Graph mining is also useful for comparative studies, allowing for the identification of distinct differences in composition of transposase associated antibiotic resistance genes in the Crohn’s disease and healthy data sets. The ability of the hybrid graph to reveal multiple genera that a given transposase sequence is present within may provide insights into the flow of horizontal gene transfer and antibiotic resistance gene spread in metagenomics samples. Graph mining is a powerful method of next generation sequencing data analysis in addition to assembly and read mapping methods.

## Methods

The Focus algorithm consists of five steps including read preprocessing, pairwise read alignment, multilevel graph set generation, multilevel graph set integration and generation of the hybrid graph, and hybrid graph trimming. Here we provide a brief overview.

1. Read preprocessor: The Focus preprocessor generates reverse complements of the input read data set and splits the reads into subsets for parallel read alignment. The preprocessor also provides options for fixed-length and quality based read trimming.
2. Pairwise read alignment: In the read alignment step of algorithm, Focus performs pairwise comparison of the read subsets generated by the preprocessor to search for potential alignments. Any overlap alignments found in the pairwise read alignment stage are used to create the initial overlap graph.
3. Multilevel graph set: The next step of the algorithm is the construction of the multilevel graph set. In this step, Focus uses heavy edge matching and node merging to create a set of graphs G_0_, G_1_ … G_n_representing increasingly coarser levels of information granularity.
4. Hybrid graph: In the fourth step, Focus backtracks through the multilevel graph set starting with the most reduced graph G_n_ to select nodes that have been determined to be the best representatives of their corresponding read clusters by local assembly analysis. These representative nodes are used to construct a hybrid graph set G^’^_n,_ G^’^_n-1_ … G^’^_0_ where G^’^_0_ contains all of the representative nodes selected and integrated from the multilevel set G_n,_ G_n-1_ … G_0_ . We call G^’^_0_ the hybrid graph.
5. Hybrid graph trimming: The hybrid graph G’_0_ is processed with a graph-filtering algorithm to remove transitive edges and nodes whose corresponding read clusters assemble into contigs that are contained in or are identical to other contigs represented in the hybrid graph. The final trimmed hybrid graph G^’^_0_ provides a concise but highly accurate and feature rich representation of the structure of the read data set [22].

**Fig. 2:**
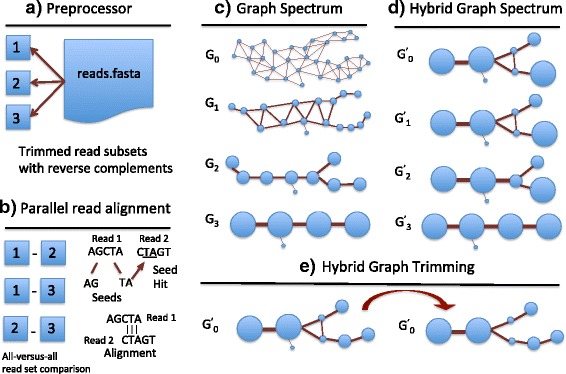
Focus assembly and analysis pipeline general overview. **a** Read preprocessor. The 5’ and 3’ read ends are trimmed using quality values and/or by a fixed length specified by the user. Reverse complements of reads are generated and the processed read data set is split into subsets for processing by the parallel read aligner. **b** Parallel pairwise read alignment. The reads subsets are pairwise aligned using a suffix-array seed based search and extend method. **c** Multilevel graph set. Iterative heavy edge matching and node merging is used to create a set of graphs. **d** Hybrid graph. Best representative nodes are selected at each graph level using partial assembly to create the hybrid graph G_0_. **e** Hybrid graph trimming. Transitive edges and redundant nodes are trimmed from G^’^_0_. Figure 2 (a-e) are a simplified overview, please see Figs. 3 and 4 and corresponding methods section for more details regarding the construction of the multilevel graph set, hybrid graph, and trimming of the hybrid graph

Once we have obtained the trimmed hybrid graph G^’^_0_ for a read data set we assign Shannon’s index scores to each node to reflect local regions of sequence diversity. A simplified overview of our workflow for the Focus algorithm can be found in Fig. 2. This methods section is organized into five subsections describing each assembly step in detail with figures, followed by a subsection describing the graph mining techniques used in this paper.

### Read preprocessor and input format

Focus accepts both fasta and fastq formatted reads. Focus requires the user to specify the number of subsets to divide the read file into for parallel read alignment. Once Focus receives the input reads and specified number of subsets, it generates the reverse complements of the input reads. The preprocessor also includes both fix-length and quality based read trimming. While generating the reverse complements, the preprocessor will first trim the 5’ and 3’ ends of each read and the corresponding 3’ and 5’ ends of its generated reverse complement with fixed lengths *l*_1_ and *l*_2_ respectively that have been provided by the user. We provide this option so that the user can remove any known adapters or tags, which may or may not be the same length, present on the 5’ or 3’ end of the reads. After fixed length trimming is completed on a read, the preprocessor will then apply quality based trimming to its 3’ end and to the corresponding 5’ end of its generated reverse complement. Given a user-provided window length of *w* and minimum average quality value *q*, the preprocessor will slide the window from the 3’ end to the 5’ end of the read until the average quality value of the window is greater than *q*. The read will be trimmed from the right endpoint of the sliding window to its 3’end. Following read trimming, the input reads and their generated reverse complements are divided evenly into the specified number of subsets. The reads in the subsets are then concatenated and indexed by a succinct dictionary structure [33]. In this structure, each nucleotide and corresponding quality value are compressed into a single byte. The read subsets are now ready for processing by the parallel read aligner.

### Parallel pairwise read alignment

The read aligner processes pairs of read subsets at a time. One of the read subsets R_q_ is designated as the query subset and the other R_r_ is designated as the reference read set. The reference read subset R_r_ is indexed by a suffix array [34] to facilitate the search for short seed matches shared between reads. Each read in R_q_ is visited sequentially and is scanned with a window of size *k* at step size *w* specified by the user to generate k-mer seeds. These seeds are used to query the reference read data set for exact matches. These exact matches are used to seed a banded Needleman alignment between the query read and reference reads. If an overlap relationship meeting user criteria for identity and length is found, the query read and reference read ids, overlap length, and overlap identity are recorded for the construction of the initial overlap graph. This process can be conducted in parallel, with different pairs of read subsets being sent to multiple processors for independent read alignment.

### Multilevel graph Set

**Fig. 3:**
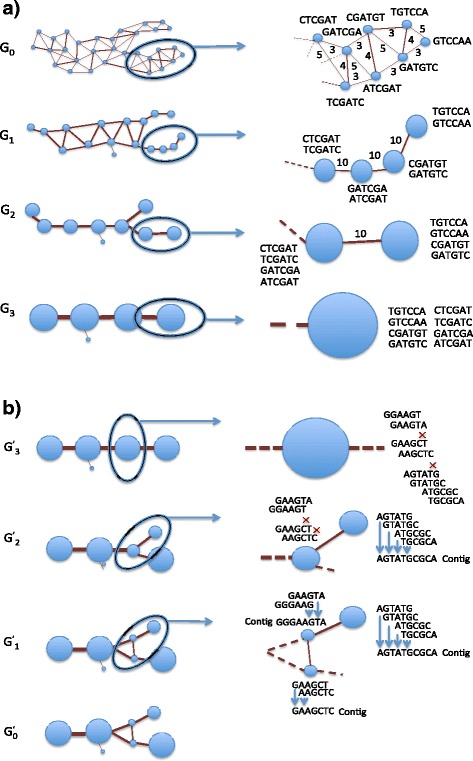
Multilevel graph set and hybrid graph. **a** Multilevel graph set. G_0_ is the most granular graph created from all of the read overlap relationships generated during read alignment. Each read is assigned to a node in G_0_ and overlap relationships are assigned to edges. Weights on edges reflect the length of the overlap relationship. Heavy edge matching and node merging is applied to create a spectrum of graphs. Clusters of reads are formed as nodes are merged at each graph level. **b** Hybrid graph. Starting with the simplest graph, in this case G_3_, Focus attempts to assemble the read clusters represented by each node. If the reads assemble into a single contig, then their corresponding node is selected as that cluster’s best representative. All nodes that are selected by Focus in G_3_ as well as nodes not selected are used to create G^’^_3_ of the hybrid graph spectrum. If a node is not selected in G_3_, then its children nodes in the next graph level, in this case G_2_, will be evaluated. The graph G^’^_2_ will be created from the nodes selected from G_3_ and G_2_ as well as from the nodes that were not selected in G_2._. We denote the final graph G^’^_0_ as the hybrid graph as it will contain all of the best representatives from G_n_ … G_0._ In (**b**), graph level integration of G_3_ … G_1_ better represents a split in the overlap graph

The initial overlap graph constructed from the read overlaps produced during the parallel read alignment process may be extremely large if there are several hundreds of thousands to several millions of reads represented in the overlap graph. This would make the data mining process very difficult, as the resulting graph would be very complex. Heavy edge matching and node merging is applied to reduce the overlap graph, creating multiple graph levels representing different levels of granularity. This section describes the multilevel graph set construction in greater detail. An illustration of this process can also be found in Fig. 3. First, parallel merge sort orders the initial edges produced by the alignment algorithm by query read id; edges with the same query read id are ordered by descending overlap length. Any duplicate edges with the same edge points as other edges in this sorted edge list are removed during the merge-sort process. This edge list is loaded by a graph data structure. For more information regarding the time-space complexity of the multilevel graph set construction and implementation of the foundational data structures used by Focus please see [35]. This initial overlap graph is denoted as G_0._ In this graph, each node represents a single read. An edge between two nodes in this graph represents an overlap relationship between their corresponding reads. Each edge in G_0_ maintains the overlap length and identity score of its corresponding read overlap relationship. The weight of the edges in G_0_ is defined to be the overlap length. This graph is the least reduced and most granular graph in the graph set G_0_, G_1_ … G_n_ and is the foundation on which the other graphs in the set are built. Each node in G_1_, G_2_ … G_n_ represents a cluster of nodes in G_0_. Two values are recorded for each node in the multilevel graph set to reflect the characteristics of its corresponding cluster in G_0_. The cluster node weight of a given node in G_1,_ G_2 …_ G_n_ is the number of nodes belonging to its corresponding cluster in G_0_. The cluster edge weight of a node in G_1_, G_2_ … G_n_ is the sum of the weights of the edges induced by the nodes in its corresponding cluster in G_0_. The nodes in G_0_ are assigned a cluster node weight of one and a cluster edge weight of zero since each node in G_0_ corresponds to an individual read.

Heavy edge matching and node merging are used to create the multilevel graph set. The heavy edge matching heuristic [36] forms a maximal matching with preference for edges with larger edge weight by matching each node v_i_ to an adjacent unmatched node neighbor v_j_, such that the edge (v_i,_ v_j_) has the largest edge weight in the set of edges incident to v_i_ that are not already part of the matching. Focus employs a modified heavy edge matching scheme to reduce the overlap graph. During the heavy edge matching process, the graph is iterated over in a user-defined number of passes such that all nodes in the graph are visited and nodes with larger maximum edge weights are visited in earlier passes. Let v_i_ be a node that the algorithm is currently visiting. The algorithm will iterate through the edges of v_i_ in the order of decreasing edge weight v_i_ to find a potential match. Let v_j_ be a node adjacent to v_i._ If v_j_ has not been matched to any previous node, the algorithm will examine the edge (v_i_, v_j_) to see if it meets user-defined thresholds, discussed next, for inclusion in the heavy edge matching. First it examines the weight of the edge, which in G_0_ is defined as the overlap length. If the weight of (v_i_, v_j_) does not meet user requirements for minimum edge weight, then the search through the edges of v_i_ is terminated and v_i_ is left unmatched. If the weight of (v_i_, v_j_) is greater than the user defined threshold for minimum edge weight, then v_j_ passes the first test. The second threshold is the density of the super node v_z_ that would result from the merging of v_i_ and v_j._ The density of v_z_ is defined as follows.

$$ \begin{array}{c}\hfill density\left({\mathrm{v}}_{\mathrm{i}},\ {\mathrm{v}}_{\mathrm{j}}\right) = density\left({\mathrm{v}}_{\mathrm{z}}\right)=\frac{\left(ew\left[{v}_i\right]+ew\left[{v}_j\right]+w\left({v}_i,{v}_j\right)\right)}{\left(\left(nw\left[{v}_i\right]+nw\left[{v}_i\right]\right)\ast \left(\left(nw\left[{v}_i\right]+nw\left[{v}_j\right]\right)-1\right)\right)/2}\hfill \\ {}\hfill =\frac{2\ast \left(ew\left[{v}_i\right]+ew\left[{v}_j\right]+w\left({v}_i,{v}_j\right)\right)}{\left(nw\left[{v}_i\right]+nw\left[{v}_j\right]\right)\ast \left(\left(nw\left[{v}_i\right]+nw\left[{v}_j\right]\right)-1\right)},\hfill \end{array} $$

where *ew* is the cluster edge weight, *nw* is the cluster node weight, and *w* is the weight of the edge (v_i_, v_j_). Here the density is the summed weights of the intra-cluster edges of the cluster in G_0_ represented by v_z_ divided by the total number of potential edges in that cluster if it was complete. This parameter controls the compactness of the merged cluster and ensures that many of the reads represented by that cluster overlap with one another. If the density of the super node that would be produced by merging v_i_ and v_j_ is greater then the user-provided threshold, then v_i_ is matched to v_j_. If the minimum threshold is not met, then the search through the edges of v_i_ for a node neighbor that meets the minimum overlap and density thresholds continues. If none are found, then v_i_ remains unmatched.

After the matching process is completed on G_0_, nodes that are a part of the matching are merged to their selected partners to form super nodes in the graph G_1_. Nodes that were unmatched in G_0_ are also mapped to new nodes in G_1_. Edges that were selected during the matching process are removed in G_1_ since their endpoints are merged into a single super node. Any parallel edges in G_1_ are combined into a single edge and their edge weights are added together. As follows, each edge in the multilevel graph set will represent the summed weight of the inter-cluster edges of the clusters in G_0_ represented by the endpoints of that edge. Heavy edge matching and node merging is applied on G_1_ to produce G_2_. This process continues until the ratio of nodes matched to graph size falls beneath a user threshold, producing a multilevel set of graphs G_0_, G_1_ … G_n_. The graph G_n_ is used to relabel the nodes in G_0_ to form a new overlap graph G_final_: any nodes co-occurring in a cluster represented by a super node in G_1_, G_2_ … G_n_ will be consecutively labeled in G_final._ This allows the nodes in G_final_ belonging to a cluster represented by a super node in G_1_, G_2_ … G_n_ to be loaded into memory concurrently by the algorithm for processing.

### Hybrid graph

After the graph coarsening process is completed, the algorithm will have produced a graph set G_0_, G_1_ … G_n_ representing the original read data set at different levels of information. However, not all sequence regions will be best represented at all graph levels. A node from a reduced graph found later in the multilevel graph set might be sufficient for representing a simple unique genomic region. In contrast, more complex genomic structures might be better represented by the more detailed graphs earlier in the graph set. For example in Fig. 3a, a branch point in original overlap graph is over reduced in graph G_3_ in the multilevel graph set. However, this branch point is captured at more granular graph levels. To address this issue, best representative super nodes are selected and integrated from multiple graph levels to create a new hybrid graph that is a highly concise yet accurate representation of the input data set. This section describes how a hybrid graph set G^’^_0_, G^’^_1_ … G^’^_n_ is constructed from the multilevel graph set G_0_, G_1_ … G_n_. The algorithm creates the hybrid graph set by selecting best representative super nodes from the original multilevel graph set beginning with G_n_ and iterating to G_0._ A best representative super node is defined as a node selected from the most reduced graph level as possible whose corresponding cluster of reads assemble into a single contiguous contig. If a read cluster does not assemble into a single contig, it might not be well represented by its current graph level. Backtracking to earlier graph levels may provide better node representatives of the reads in that cluster. To select the best representatives, the algorithm first iterates through G_n_. For each super node in G_n_, its corresponding cluster subgraph in G_final_ is loaded into memory. Focus employs graph-cleaning techniques first introduced by [37] and used commonly by many assembly tools. Short dead-end branches that are shorter than a user provided threshold are removed from the subgraph. Small bubbles in the graph, which are two distinct paths in the graph that have the same beginning and ending nodes, are also removed by eliminating the least weighted path. The subgraph is then transitively reduced following the approach in [16]. If the resulting graph is a single path representing a contiguous contig, then the super node is selected as the best representative of that read cluster. The read cluster is assembled into a contig and recorded on file. After the iteration through the nodes of G_n_ is complete, all selected best representatives are mapped to nodes in G^’^_n_. Nodes that were not selected as best representatives are also mapped to nodes in G^’^_n_. After the best representative selection on G^’^_n_ is complete, the algorithm begins the super node iteration and assembly evaluation process on G_n-1_. If a node in G_n-1_ is a component node of a merged super node in G_n_ that was previously selected by the assembly algorithm as a best representative, it will not be evaluated or included in G’_n-1_ since its parent was already chosen as the best possible representative. The graph G^’^_n-1_ is created from all of the best representatives selected from G_n_ and G_n-1_ as well as from the nodes that were not selected in G_n-1._ Contigs assembled from the best representatives in G_n-1_ are recorded to file. The graph G^’^_n-2_ will be composed of best representative nodes selected from G_n_, G_n-1_, G_n-2_ and the nodes that were not selected in G_n-2_. This process is continued for G_n-3_ … G_0._ The final graph G^’^_0_ will contain all best representatives selected from G_n_, G_n-1_ … G_0_. We call this graph the hybrid graph since it is the integration of all graph information levels. As in the multilevel graph set, each edge in the hybrid graph set represents the summed total of the edge weights of the inter-cluster edges of the two clusters in G_final_ corresponding to the endpoints of that edge. Please see Fig. 3b for an example and [22] for more algorithmic details regarding the construction of the multilevel graph set and hybrid graph set.

### Hybrid graph filter

**Fig. 4:**
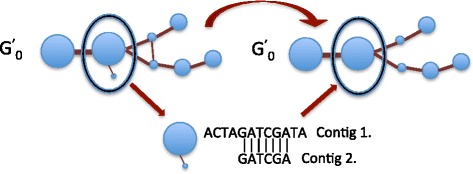
Hybrid graph trimming. For each node, if its corresponding contig can be mapped to a neighboring node’s contig, then that node is removed from the hybrid graph. Transitive edges are also removed from the hybrid graph

Once the hybrid graph G^’^_0_ is created, it is filtered to remove any redundant nodes whose corresponding contigs are contained within other contigs represented in the hybrid graph. For each node in G^’^_0_, the graph-filtering algorithm will load its corresponding contig into memory. If the length of the contig is less then a user provided threshold, then the filter will load each adjacent node’s contig into memory. The current contig is aligned against its neighboring contigs. If the current contig can be mapped to any of its neighboring contigs, then its corresponding node along with its incident edges will be removed from the hybrid graph as shown in Fig. 4. Any transitive edges in the hybrid graph are also removed. After the filtering algorithm is complete, each node in the hybrid graph will represent either a homologous region shared between species, a sequence repeat, or a unique genomic region.

### Hybrid graph data-mining

The hybrid graph is used for mining and extraction of biologically significant features, since it provides the most concise, yet accurate structural view of the read data set that could be obtained from integrating the multilevel graph set. In this graph, the degree of a given node can provide much information about the characteristics of the sequence region from which its corresponding reads were derived. If a node has a single pair of in and out edges, it is possible that this node is from a uniquely represented genomic region. In contrast, if a node has several in and out edges, this might indicate that the node represents a sequence region that is repeated throughout a genome or is shared between multiple species. The number of edges incident to a node might reflect the number of diverse sequences that its corresponding genomic region is present within.

**Fig. 5:**
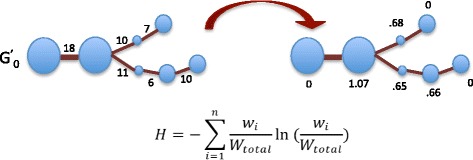
Shannon’s index scores. Calculation of Shannon’s index scores. Notice that nodes with a greater degree have a higher Shannon’s index score

**Fig. 6:**
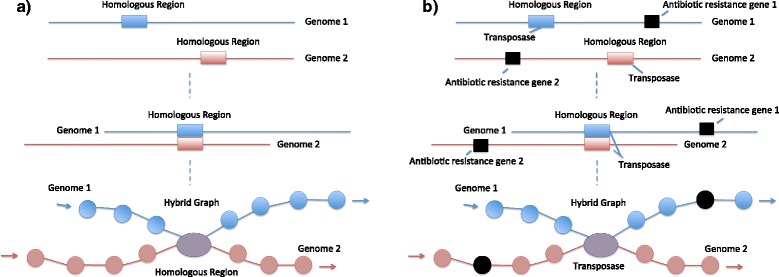
Genomic features and related graph structure. **a** Genomes one and two share a region of sequence homology. In the hybrid graph this homologous region will be reduced to a single node (purple). Two paths representing the unique regions in genomes one and two preceding the 5’ end of the homologous region enter the reduced node. The two paths exiting the node represent the unique genomic regions in genomes one and two following the 3’ end of the homologous region. Blue represents genome one and red represents genome two. Observe that we can identify which species a given region is present in by analyzing its representative node’s neighbors in the hybrid graph. For example, obtaining species level classification for the nodes adjacent to purple node would identify in which species the region represented by the purple node was present. **b** Genomes one and two share a homologous transposase sequence. Each of these genomes also contains a transposase associated antibiotics resistance gene. As in (A), the homologous region containing the transposase sequence is reduced to a single node shown here in purple. The nodes corresponding to the antibiotics resistance genes are colored black and blue represents genome one and red represents genome two. Graph exploration of the node neighborhood of the node representing the homologous transposase sequences will reveal antibiotics resistance genes associated with them

The first aim of this manuscript is to show that repetitive and mobile elements are associated with node characteristics. Shannon’s index is very popular for measuring biological diversity [38], however; it has not yet been applied for characterizing sequence diversity captured by graph structures in assembly graphs. Shannon’s index encompasses both the edge richness and edge weight evenness of a given node. Edge richness refers to the number of edges incident to a node. Edge evenness measures the distribution of weight across the edges. The formula for Shannon’s index is given by

$$ H=-{\displaystyle {\sum}_{i=1}^n\frac{w_i}{w_{total}} \ln \left(\frac{w_i}{w_{total}}\right),} $$

where *n* is the number of incident edges, *w*_*i*_ is the weight of the *i*th edge, and *W*_total_ is the total weight of all incident edges. As seen in Fig. 5, a greater number of edges and an equal distribution of edge weights increases a node’s Shannon index score. The maximum Shannon’s index score that can be assigned to a node *v* is ln(*n*), where *n* is the number of edges incident to *v.* The score of a node that has two edges with similar large weights and multiple edges with very small weights will not be very different from the score of a node with only two edges with similar weights. Thus any possible spurious edges with small edge weights relative to the edge weights of the other incident edges will not greatly impact a node’s Shannon index score. In the results section, it is demonstrated that repetitive and mobile elements are associated with graph structure that is captured by the Shannon’s index. Figure 6a provides an example illustrating homologous regions shared between two genomes and corresponding graph structure.

The second aim of this paper is to extract transposase genes that are present in multiple sequence regions and to identify which genera they are distributed in. Antibiotic resistance genes associated with these transposase sequences are also mined from the hybrid graph. In this section, it is discussed how, for each given transposase gene present in multiple sequence regions, the hybrid graph can be used to identify which genera the transposase gene is distributed in. This section also discusses how the hybrid graph can be used to obtain transposase associated antibiotic resistance genes through local exploration in the hybrid graph. Observe that in Fig. 6a, the distribution of genera that a transposase sequence is shared across can be obtained by taxonomically classifying the sequences of the adjacent node neighbors of its corresponding node in the hybrid graph. Similarly, any antibiotics resistance genes that are associated with a given transposase sequence can be found by exploring the graph locally around its corresponding node. Figure 6b provides an example of how local graph exploration can reveal antibiotics resistance genes associated with transposase sequences.

## Results

In this section the distribution of transposase genes and associated antibiotic resistance genes across bacterial genera in the gut microbiomes of healthy individuals and individuals with Crohn’s disease is characterized using graph mining techniques. To achieve this goal this study has been divided into three specific aims discussed previously in the background.

1. Demonstrate that repetitive and transposable elements are associated with node characteristics
2. Identify and characterize the phylogenetic distribution of antibiotic resistance gene classes associated with transposase/integrase sequences in healthy individuals and individuals with Crohn’s disease.
3. Perform a competitive assembly evaluation of the assembler against other well-known assembly tools.

The results are divided into four sections. First, a general overview of the data sets and an analysis of the distribution of genera present in the Crohn’s disease and healthy data sets are provided. This study was conducted to evaluate the characteristics of the data sets in the context of previous research. Statistically significant differences were found in the relative abundances of prevalent genera in the Crohn’s and healthy data sets.

Second, aim 1 is addressed. For aim 1, it is shown that a greater proportion of nodes annotated with repetitive and mobile elements are assigned high Shannon’s index scores compared to nodes annotated with other gene categories. First an analysis and discussion regarding the distribution of Shannon’s index scores across the nodes of the hybrid graphs of the thirteen data sets is presented. This is followed by briefly exploring the characteristics of features associated with nodes with high Shannon’s index scores. The most common blastx hits to the NCBI blast database [39] for extremely high scoring nodes (the two highest scoring nodes for each data set) were to transposases and integrases (33.3 % of all predicted genes). We then used gene and rRNA operon predictions, SEED subsystems [40], and ACLAME library [41] to examine biological features associated with the remaining graph nodes. Nodes assigned with transposase/integrase genes and rRNA operon DNA had a greater proportion of high Shannon’s index scores in comparison to nodes assigned with other gene categories from the SEED subsystems (q = 2.44 × 10-04; paired Wilcoxon tests).

Third, addressing aim 2, a comparative study of antibiotics resistance genes associated with transposase/integrase sequences present in multiple sequence regions in the Crohn’s and healthy data sets was conducted. In aim 1, it is demonstrated that a greater proportion of nodes annotated with mobile genetic elements and rDNA operons had high Shannon’s index scores compared to nodes annotated with other gene categories. For aim 2, transposase/integrase sequences found on nodes with high Shannon’s index scores are analyzed since they are likely to be present in multiple sequence regions. We identify all high degree nodes with Shannon’s index scores greater than one that had hits to transposases, identify which genera their corresponding contig sequences are present in, cluster the transposases according to their phylogenetic distribution, and determine if sequence regions associated with the transposases in the resulting clusters are enriched for antibiotic resistance genes. The transposase nodes in the Crohn’s data sets clustered into twenty sets and the nodes in the healthy data set clustered into ten sets. For each of these clustered sets, predicted genes in associated contigs were extracted and DIAMOND [42] was used to align the predicted genes to the CARD database of antibacterial resistance genes [43]. Fisher’s exact test with FDR corrected p-values was applied to determine if any clusters were enriched with classes of antibiotics. Several of the transposase clusters generated in the Crohn’s disease and healthy control data sets were enriched with various classes of antibiotic resistance genes. This comparative study provides insight into the differences in the distribution and species composition of resistance genes in healthy individuals and Crohn’s patients, whose disease is associated with gut microbiome perturbation [44] and is often treated with antibiotic regimens for secondary complications such as bacterial overgrowth and abscesses [31].

Finally the results are concluded by a competitive assembly evaluation of Focus against metagenomics assemblers, IDBA-UD, Omega, and MetaVelvet.

### Data sets

Thirteen data sets were downloaded from the NCBI sequence read archive [45]. Five of the data sets were sequenced from the gut microbiome of individuals with Crohn’s disease and eight of the data sets were sequenced from the gut microbiomes of healthy individuals. Table 1 shows the subject ids for each data sets and their phenotype information. Table 1 also displays the number of reads in each data set prior to read trimming as well as the number of processed reads produced by the Focus read preprocessor, which includes generated reverse complement reads. The Focus preprocessor was set to trim 20 bps off of the 5’ read ends and 50 bps off of the 3’ read ends to remove tags and adaptors. The minimum quality value for the quality based trimming was set to 25. Any read whose length fell below 75 bps was discarded from the processed data set. A hybrid graph was constructed for each individual data set.

**Table 1:** Data set characteristics

Subject ID	Phenotype	Sample	Runs	Total Reads	Processed Reads	Mapped (%)	Shannon’s Index
33	Female, Crohn’s	SAMN00829176	SRR49544 SRR497943 SRR497952	1775071	3478940	80.6 %	1.91
58	Female, Crohn’s	SAMN00829163	SRR497643 SRR497648 SRR497650	2049784	4025328	68.4 %	1.64
92	Female, Crohn’s	SAMN00829171	SRR497646 SRR497657 SRR504939	1950395	3848348	61.4 %	1.73
104	Male, Crohn’s	SAMN0082172	SRR497946 SRR497948 SRR497949	2175693	4284474	72.1 %	1.97
68	Male, Crohn’s	SAMN00829168	SRR497645 SRR497652 SRR497654	2084020	4113996	79.0 %	1.49
763820215	Female, Healthy	SAMN00078732	SRR063543 SRR063544 SRR063545	2395215	4744426	88.9 %	0.59
764042746	Female, Healthy	SAMN0036587	SRR063587 SRR063588 SRR063589	2260051	4463710	81.5 %	0.56
809635352	Female, Healthy	SAMN00043742	SRR063903 SRR063904	2820502	5533454	64.0 %	1.33
638754422	Female, Healthy	SAMN0075991	SRR061730 SRR061731	2944584	5823782	77.7 %	0.96
764143897	Female, Healthy	SAMN00071891	SRR063539 SRR063548 SRR063549	2496427	4945024	70.6 %	1.27
604812005	Male, Healthy	SAMN0006554	SRR063905 SRR063906	2680706	5287590	79.6 %	0.75
763435843	Male, Healthy	SAMN00037012	SRR063553 SRR063554 SRR063555	2513710	4962822	73.1 %	1.11
763961826	Male, Healthy	SAMN00040248	SRR063583 SRR063584 SRR063585	2436744	4798677	77.4 %	0.98

**Fig. 7:**
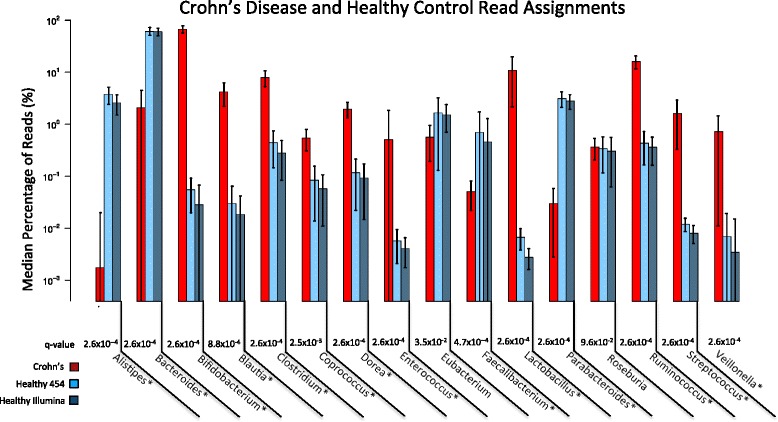
Taxonomic read classification. Median percentage of reads assigned to major genera present in the Crohn’s disease and healthy read data sets. A ‘*’ denotes a significant difference in the median read percentages in the Crohn’s and healthy samples

For the purpose of examining the characteristics of the read data sets, the BWA [46] aligner was used to align the sequence reads against the Human Microbiome Project microbiome reference sequences [47]. Each read was classified to a genus by its best alignment hit (Additional file 1). Table 1 displays the percentage of reads that could be mapped back to a reference genome for each data set. Figure 7 shows the median read percentages assigned to highly abundant genera that at least 0.5 % of reads were assigned to in at least three samples. This threshold was selected to eliminate low abundance genera as well as genera that were highly abundant in only one or two individuals. We also downloaded Illumina data sets sequences from the same set of healthy individuals to show that the genera distribution in the samples is consistent. Figure 7 shows that the median percentage of reads for highly abundant genera is very similar between the Illumina and 454 read data sets, providing confidence that the sequence process was able to correctly capture the abundance ratios. Figure 7 also shows distinct differences in the abundances of major genera present in the Crohn’s and healthy individuals with statistically significant decreases in *Alistipes*, *Bacteroides*, *Faecalibacterium*, and *Parabacteroides* in Crohn’s disease samples. The genera *Bifidobacterium*, *Blautia*, *Clostridium*, *Coprococcus*, *Dorea*, *Enterococcus*, *Lactobacillus*, *Ruminococcus*, *Streptococcus*, and *Veillonella* were significantly increased in Crohn’s disease samples. The Mann–Whitney U test was used to calculate p-values. Previous studies have found a wide range of alterations in the microbiome of Crohn’s disease patients versus healthy individuals [48]. Examples of frequent shifts found previously in Crohn’s disease microbiota composition are decreases in *Faecalibacterium prausnitzii,* increases in *Ruminococcus gnavus*, and increases in *Enterococcus faecium* [49–52]. The consistency between Illumina and 454 data sets and observations of microbiota shifts found in previous literature provides evidence that our selected data sets provide an appropriate view of biological differences between the microbiome of healthy individuals and individuals with Crohn’s disease.

### Repetitive and transposable elements are associated with node characteristics

#### Shannon’s index score distribution and functional gene categories

**Fig. 8:**
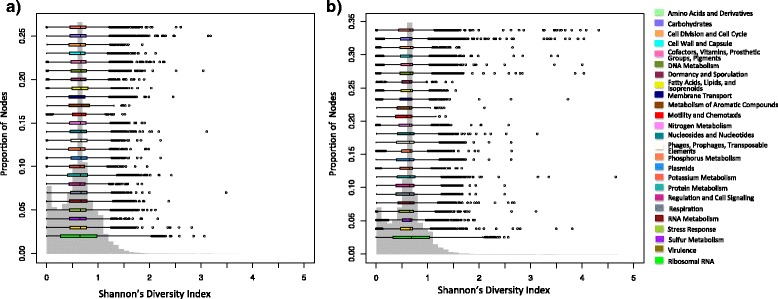
Shannon’s index score distribution and Seed subsystem assignment. Node count distribution for assigned Shannon’s index scores. Boxplots display the range of scores for predicted genes assigned to level 1 classifications in the SEED subsystems. **a** Shannon’s index score distribution of the nodes of the hybrid graphs generated from the Crohn’s disease data sets. **b** Shannon’s index score distribution of the nodes of the hybrid graphs generated from the healthy data sets. Note that the highest node counts and predicted genes fall into the same score range for both sample types

This section provides an overview of the distribution of Shannon’s index scores found across the nodes in the hybrid graphs of the Crohn’s disease and healthy data sets. Figure 8 displays the distribution of node counts for the Shannon’s index scores. Notice that the Shannon’s index scores that have the greatest node counts fall in the range of .6 to .7. If a node had a single in and out edge representing a single unique path and the in and out edges were evenly weighted, then its corresponding Shannon’s score would be ln(2)≈.69. Thus, nodes whose corresponding sequence is a unique genomic region will have Shannon’s index scores in this score range.

The SEED [41] is an organizational database system that provides five levels of hierarchical gene functional categorization with the first level being the most general level of classification. The FigFams, which form the leaves of this hierarchy, are sets of proteins that share the same function and are similar at the sequence level. FragGeneScan [53] was used to predict genes in contigs for all data sets. We downloaded the SEED protein database and used DIAMOND, which was chosen because of its scalability to large data sets and similar degree of sensitivity as BLASTX, to align the predicted genes to the SEED FigFams at a 40 % identity threshold. The SEED subsystems database was used to assign each gene to a level 1 functional categorization if possible. Most of the predicted genes were located on contigs whose corresponding nodes fell into the .6 - .7 score range as well, as shown by Fig. 8. However, there are many outlier genes that have a much greater Shannon’s index score, indicating that they might be found on contigs whose nodes represent repetitive sequence or sequence that is shared between two or more species. In the following section, we first provide a brief characterization of the most extreme outlier genes, showing that many of these genes are transposase and integrases. We then demonstrate that nodes annotated with repetitive and mobile genetic elements have a greater proportion of high Shannon index scores compared to nodes annotated with other gene categories.

#### Characterization of biological features on outlier nodes

Here we briefly examine the biological features on nodes with the most extreme Shannon’s index scores. The two highest scoring nodes in each data set that had at least one edge with minimum edge weight of 5000 were obtained from the hybrid graphs. The minimum edge weight was set to filter low coverage nodes in the data set. Blastx against the NCBI non-redundant protein database was used to identify biological features on the contigs corresponding to the selected nodes. Table 2 displays the results of the feature hits found on the contig sequences. The most frequent hits that were not to hypothetical or uncharacterized proteins were to transposase and integrase related elements. A total of 33.3 % of the hits were to transposases and integrases.

**Table 2:** Sequence features found on nodes with the highest Shannon’s index scores

Sample	Shannon's Index Score	Sequence Feature(s)	Blast E-Values
Female 33	3.69	Transporter, RelB/DinJ, Transposase	5e-15, 5e-32, 4e-32
Female 33	3.66	Transposase	1.00e-45
Female 58	2.58	Hypothetical protein	6.00e-04
Female 58	2.46	TonB-dependent receptor	6.00e-51
Female 92	2.82	Delta-lactam-biosynthetic de-N-acetylase	3.00e-57
Female 92	2.74	Resolvase	4.00e-87
Male 104	2.53	Transposase, Cbl	3e-32, 7e-27
Male 104	2.47	Phosphatase, Histidine phosphotransferase	2e-108, 4e-72
Male 68	2.86	PG1 protein	1.00e-32
Male 68	2.43	Transposase	1.00e-45
Female 638754422	3.75	Transposase, IS4 family	8.00e-61
Female 638754422	3.68	Transposase	2.00e-22
Female 763820215	3.26	Major facilitator transporter	0.00e + 00
Female 763820215	2.51	ATPase AAA	1.00e-120
Female 764042746	2.69	Transposase	1.00e-34
Female 764042746	2.64	DEAD/DEAH box helicase	1.00e-172
Female 809635352	4.65	30S ribosomal protein S12	1.00e-26
Female 809635352	4.58	Uracil phosphoribosyltransferase	7.00e-05
Female 764143897	3.90	None	NA
Female 764143897	3.63	None	NA
Male 604812005	2.96	Tetratricopeptide repeat protein	6.00e-47
Male 604812005	2.71	ATP-dependent DNA helicase RecQ	2.00e-149
Male 763435843	3.65	Putative transposase, Major Facilitator Superfamily protein, Glycosyltransferase, Group 1 family protein	5e-12, 1e-24, 8e-13
Male 763435843	3.47	Transposase	1.00e-43
Male 763961826	2.72	None	NA
Male 763961826	2.65	Transposase family protein, DNA polymerase IV	4e-67, 2e-51

#### Selection of a threshold for high Shannon’s index scores

In the previous section we examined the biological features on a small subset of nodes with extreme Shannon’s index scores. Next, we demonstrate that nodes annotated with repetitive and mobile elements have a greater proportion of high Shannon’s index scores. However, minimum threshold for a Shannon’s index score to be considered high must be defined. Recall that for a given node with *n* edges, the maximum Shannon’s index score that can be assigned to that node is ln(*n*). An appropriate threshold will exclude nodes that possess a single entering and exiting edge as these nodes might be more likely to be part of unique genomic region. The minimum threshold that would eliminate these nodes is ln(2) ≈ .69 as this is the maximum Shannon’s index score that could be assigned to a node with two edges. However, a node could possess two evenly weighted edges and a third spurious edge that has a small edge weight, pushing this node past the minimum threshold. Thus the minimum threshold is raised to ln(3), which is the maximum score a node with three evenly weighted edges could be assigned. For the sake of simplicity ln(3) ≈ 1.1 is rounded to one.

#### Characterization of biological features on high scoring nodes

In this section, we demonstrate that nodes annotated with repetitive and mobile genetic elements have a greater proportion of high Shannon’s index scores. To achieve this, we compare the proportion of nodes assigned high Shannon index scores for each of the SEED functional categories to the proportion of nodes assigned high Shannon index scores for rRNA operon and transposase/integrase sequences.

The Meta-RNA [54] software tool was used to predict rDNA operon sequences in all of our contig sets. Meta-RNA was chosen because of its ability to detect rRNA sequences in fragmented metagenomics data. To further investigate the distribution of transposase and integrase sequences across nodes, the protein sequences of all transposases and integrases were downloaded from the ACLAME database. We used DIAMOND to align the predicted genes to the transposase and integrase protein sequences from both the ACLAME library and SEED FigFams at a 40 % identity threshold. For each read set in the Crohn’s disease and healthy control data sets, the proportion of nodes with Shannon’s index scores greater than one for each of the SEED functional categories, the rRNA operon sequences, and the transposase and integrase sequences was determined. The paired Wilcoxon test was applied to compare the high scoring node proportions for each SEED functional category pooled from the Crohn’s and healthy data sets against the pooled rRNA operon high scoring node proportions followed by the pooled transposase and integrase sequence high scoring node proportions. The paired Wilcoxon tests with FDR correction showed that both the transposases and integrases and rRNA operons had a significantly higher proportion of nodes with Shannon’s index scores greater than one than the SEED functional categories (q = 2.44 × 10^−04^; Additional file 2).

### Mining and characterization of transposase associated antibiotics resistance genes

**Fig. 9:**
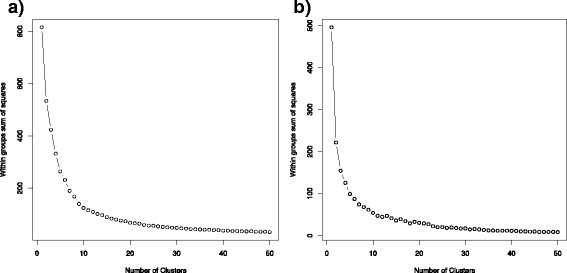
K-means clustering elbow plots. Elbow plots for the k-means clustering of the transposases nodes for the Crohn’s disease and healthy data set. The left plot (**a**) shows the within groups sum of squares for the Crohn’s disease data sets and the right plot (**b**) shows the sum of squares for the healthy data sets. The within sum of squares was much higher for the Crohn’s disease data sets versus the healthy data sets. The number of clusters for the Crohn’s disease data sets (20) and the healthy data sets (10) were chosen such that their sum of squares were roughly equivalent

**Fig. 10:**
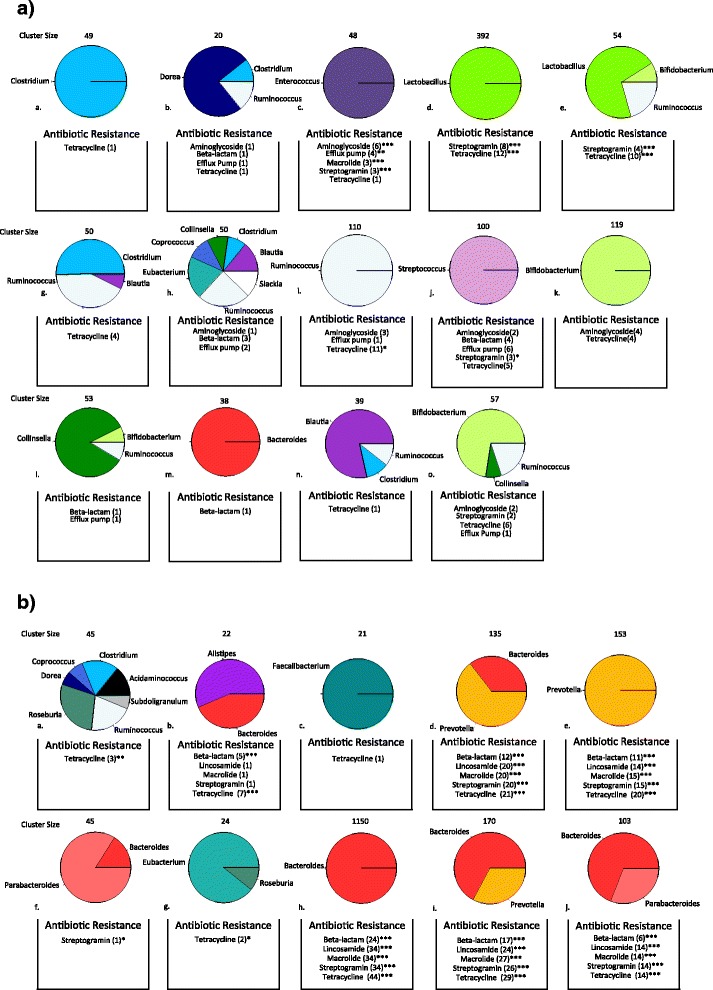
Phylogenetic clusters of transposases with antibiotic class enrichments. Transposase associated nodes were clustered using k-means clustering according to the distribution of genera that the contigs of their neighboring nodes were assigned to. **a** Phylogenetic clusters of transposases in the Crohn’s disease data sets. **b** Phylogenetic clusters of transposases in the healthy control data sets. Enrichments of antibiotic resistance gene classes for the 5-neigborhood of the transposase nodes are indicated at the .05, .01, and .001 significance level (*, **, ***)

As reviewed in the background, in addition to transposases, bacterial transposons often carry genes for antibiotics resistance allowing for the spread of antibiotic resistance mechanisms [23]. In this section, for each transposase-associated node with a Shannon’s index score greater than one, the genera of the sequences that contain that transposase are identified. For a given node, the contigs corresponding to each of the node’s adjacent neighbors in the hybrid graph are obtained. Majority read vote was used to assign each contig to a genus by the Human Microbiome Project microbiome reference sequences. If a contig could not be classified to a genus then it was classified as unknown. For each transposase-associated node, a vector *v* = (*x*_1,_*x*_2, …_*x*_*n*_) was created, where x_i_ is the summed length of the neighboring contigs assigned to genus *i* normalized by the total length of all of the neighboring contigs. K-means clustering was used to cluster the high scoring transposase nodes into groups based on the Euclidean distance of these vectors, which represent the distribution of the genus level classifications of the sequences containing each transposase region. Transposase nodes that had more than 20 % of adjacent sequence classified as unknown were not included in the clustering. Multiple iterations of k-means clustering and the generated elbow plots shown in Fig. 9 were used to select ten as the number for k for the transposase nodes from the healthy data set and twenty for the transposase nodes from the Crohn’s disease data set. For the purpose of examining the occurrence of antibacterial resistance genes among phylogenetically conserved transponsases, all of the antibacterial resistance gene protein sequences were downloaded from the CARD database. Any resistance gene tagged as a gene variant was removed from the set to avoid false positive hits. DIAMOND was used to align the predicted genes in the contigs for each data set against the antibiotic resistance gene proteins at a 90 % identity threshold. For each transposase node we extracted all of the contigs from its 5-neighborhood node set to search for hits to antibacterial resistance genes localized near transposase sequences. The 5-neighborhood of a given node is the set of nodes no further than a path distance of five from that node. Fischer’s test was used to determine if the number of hits to classes of antibiotics resistance genes in the neighborhoods of the transposase nodes was enriched in comparison to the total number of hits in the total nodes set. Figure 10 shows the phylogenetic transposase clusters for the Crohn’s disease A) and the healthy data sets B). Each pie chart displays the average distribution of the abundant genera (at least 5 % of the total composition; Additional file 3) of the contigs of the neighboring nodes of each transposase-associated node in that cluster. For each cluster we list the number of 5-neighborhood node set hits to antibacterial resistance gene classes. FDR corrected enrichments at the .05, .01, and .001 significance levels are indicated and can also be found in Additional file 4. The number of transposase-associated nodes in each cluster are listed above each pie chart. If a transposase cluster had less than twenty members, then it was not included in Fig. 10 or subsequent analysis. Also, two clusters from the Crohn’s disease data set had redundant phylogenetic distributions of highly abundant genera; the larger cluster was used for further analysis.

In the transposase clusters generated from the Crohn’s disease data sets, there were several clusters that were enriched for antibiotic resistance gene classes. In particular, there was an *Enterococcus* phylogenetic transposase cluster that was not found in the healthy control data set, shown in Fig. 10a (c). The node set obtained from the 5-neighborhood of all of the transposase associated nodes in the *Enterococcus* cluster was enriched with aminoglycoside, macrolide, and streptogramin resistance gene classes. The aminoglycoside resistance gene class was enriched at the .001 significance level and represented hits to the intrinsic *Enterococcus Faecium* aac(6')-Ii gene. The macrolide and streptogramin classes were enriched at the .01 level of significance and represented hits to the intrinsic *Enterococcus Faecium* msrC gene. A single hit to the tetracycline resistance gene class was most similar to the tet(L) gene and aligned to a *Enterococcus* plasmid. Two clusters whose transposase-associated nodes had many neighbors with contigs classified to *Lactobacillus* were also significantly enriched with antibacterial resistance gene classes, shown in Fig. 10a (d,e). The tetracycline class hits were most similar to tet(W) genes found in *Bifidobacterium*, *Lactobacillus*, and *Streptococcus.* The streptogramin class hits were to the vat(E) gene found in *Enterococcus Faecium* and some *Lactobacillus* plasmids. The *Ruminococcus* group shown in Fig. 10a (i) was enriched with tetracycline resistance genes with hits to tet(O) and tet(W).

In the healthy control data sets, resistance genes were most prevalent in transposase clusters associated with *Bacteroides* and *Prevotella.* The *Bacteroides* cluster, Fig. 10b (h) was also the largest cluster in the group. Figure 10b (d, e, h, i, j) were all enriched resistance genes from the beta-lactam, lincosamide, macrolide, streptogramin, and tetracycline resistance gene classes. The enrichments for the lincosamide, macrolide, streptogramin resistance gene classes were due to hits to the ermG and ermF macrolide-lincosamide-streptogramin B resistance proteins. The ermB, ermF, ermG, and ermS genes are common sources of resistance in *Bacteroidales* strains found in the intestine [55]. The enrichments for the beta-lactam class of resistance genes were due to hits to class A beta-lactamases which are found in strains of *Bacteroides* and *Prevotella*. Tetracycline class enrichments were from hits to the tet(Q) resistance gene, also found in *Bacteroides* and *Prevotella*. The transposase cluster associated with *Bacteroides* and *Alistipes,* Fig. 10b (b), was enriched for class A beta-lactamase and tet(Q) resistance genes. Antibiotics resistance gene hits with gene descriptions can be found in Additional file 5.

### Comparative assembly

For the purpose of demonstrating Focus’s performance as a pure assembly tool, we applied Focus and three other popular assemblers, IDBA-UD, Omega, and MetaVelvet, to four selected data sets from the healthy and Crohn’s disease individuals. Two data sets were chosen from the healthy individuals and two data sets were chosen from individuals with Crohn’s disease. These data sets had the highest calculated Shannon’s index for each of their respective groups (Table 1). Results from the comparative assembly are shown in Table 3. Statistics used to evaluate the assemblies included the number of contigs produced by each assembler, N50 statistic, and percentage of reads successfully mapped back to each assembly. The N50 length is a commonly used statistic to assess assembly quality [56]. It is the length of the longest contig such that the sum of the lengths of contigs larger or equal to the length of that sequence covers at least half of the estimated genome size. In the case that reference genome lengths are unavailable, the assembly length is often used as an approximation. Previous research has mentioned several challenges with applying the N50 statistic for assembly evaluation; particularly in metagenomics where sequence abundances vary [15] and where total assembly lengths are different between assemblers [56]. To address this issue we also include the NG50 [56], which is analogous to the N50 except the estimated genome size is used instead of assembly size. Since the reference sequences for these data sets are unknown, we sum the average genome lengths of the complete genomes available through the NCBI RefSeq for the most abundant genera, shown in Fig. 7, present in the Crohn’s and healthy data sets as a reasonable estimate of total genome length present in the data sets. The calculation of the average genome lengths and estimated total genome length can be found in Additional file 6. The estimated total genome length was calculated to be 46,498,455 bps by the above method.

**Table 3:** Comparative assembly results

Subject ID	Assembler	Number of Contigs ≥500 bps	N50 (bps)	NG50 Abundant Genera (bps)	(%) reads mapped to contigs	Chimeric contigs	Contigs with taxonomic assignment	Contigs with unknown assignemnt
33	Focus	89994	1310	2050	95.5 %	445	68037	21512
	IDBA-UD	33932	1267	1040	89.7 %	56	24534	9342
	MetaVelvet	18355	709	N/A	15.0 %	30	13250	5075
	Omega	1887	2037	N/A	12.5 %	74	156	1657
104	Focus	150930	1286	2371	95.7 %	720	102618	47592
	IDBA-UD	61848	1236	1759	87.3 %	70	42383	19395
	MetaVelvet	34011	702	513	17.2 %	41	22556	11414
	Omega	2579	2652	N/A	14.8 %	45	532	2002
764143897	Focus	159196	1599	2595	95.2 %	1383	94968	62845
	IDBA-UD	60679	1736	4009	92.1 %	265	34174	26240
	MetaVelvet	46123	723	626	30.4 %	140	24367	21616
	Omega	3433	2531	N/A	15.9 %	78	1637	816
809635352	Focus	251461	1296	2653	91.0 %	2819	127846	120796
	IDBA-UD	106962	1199	2976	85.2 %	571	51458	54933
	MetaVelvet	76809	719	774	30.7 %	269	33762	42778
	Omega	2546	2681	N/A	8.3 %	92	1815	639

The Focus and IDBA-UD assemblers performed the best on these data sets in terms of N50 length, NG50 length, and percentage of reads that were successfully mapped back to their assemblies. Read mapping was conducted with BWA. Omega had the largest N50 length; however, the percentage of reads that were successfully mapped back to contigs was very low. This indicates that Omega only assembled a very small fraction of the input data set. The size of each of the Omega assemblies was so small that the NG50 statistic could not be calculated for any of the data sets using the estimated total genome length. MetaVelvet had a smaller N50 statistic and a lower percentage of mapped reads indicating that a low percentage of the input data set was assembled into small fragmented contigs. Focus and IDBA-UD had similar N50 statistics and percentage of reads successfully mapped back to contigs. For three of the data sets, Focus had a slightly larger N50 length than IDBA. Focus had larger NG50 lengths than IDBA for two of the data sets. Chimeric contigs are contigs in which at least 25 % of the reads do not map to the genus to which the contig was assigned. Results in Table 3 show that each assembler had a very small fraction of detectable chimeric contigs. A large number of the contigs produced by each assembler could not be assigned to any genus. These results demonstrate that Focus is capable of producing assembly results that are competitive with and exceed existing tools.

## Discussion

We have developed a novel graph mining and assembly algorithm that is capable of extracting useful biological information and producing high quality assembly results. Our algorithm captures genome structural information using a hybrid graph. The initial overlap graph is incrementally reduced using heavy edge matching and node merging to create a graph spectrum, G_0_, G_1_, … G_n_ that represents a read data set at multiple levels of information. To provide the most accurate yet succinct representation of the input data set, nodes from each graph level are selected as best representatives of their corresponding read clusters and combined into a single hybrid graph G^’^_0_. Each node in this graph represents either a unique region, repetitive element, or region conserved between multiple species. We assigned a Shannon’s index score to each node to numerically describe the number of incident edges and the evenness of their weights. We show that repetitive elements, in particular rRNA operons and transposase genes, are associated with higher Shannon’s index scores. We then extract transposase genes whose corresponding nodes had high Shannon’s index scores in five read data sets obtained from the gut microbiome of individuals with Crohn’s disease and eight read data sets obtained from the gut microbiome of healthy controls. We clustered the resulting transposase genes into groups determined by the distribution of genera that the contigs obtained from the adjacent neighbors of their corresponding nodes were classified too. We then test for enrichment of antibiotic resistance genes in the 5-neighborhood of the nodes in each transposase cluster. Distinct differences were apparent in the Crohn’s disease and control data set clustering results. An enterococcal transposase cluster that was enriched with various antibacterial resistance gene classes was present in the Crohn’s disease clustering results while being absent from the healthy control clustering results. *Enterococcus* species are often overrepresented in Crohn’s disease data sets. Other sources of antibacterial resistance genes were from *Lactobacillus* associated transposase clusters. Origins of antibiotic resistance in healthy individuals were heavily biased towards *Bacteroidales* species. The distribution of the number of transposases was relatively even across the Crohn’s disease clusters, while in the healthy disease data sets most transposases were found in a *Bacteroides* associated cluster.

This paper highlights the ability of the assembly graph to be a powerful data-mining support that can capture meaningful biological information and patterns in its structural features. Our graph theoretic model is concise yet feature rich, allowing for the efficient detection of biologically meaningful graph structures. We foresee the expansion of our model and the development of novel domain-specific graph mining techniques for other next generation sequencing applications. For example, in cancer research genomic rearrangements, copy number variations, and fusion genes are prevalent [57]. These biological features are likely to be reflected in the structure of the assembly graph for input data sets. We are also exploring further applications of our model for metagenomics data, such as graph-based read filtering of target species from metagenomics samples.

## Conclusions

In conclusion, we have developed a powerful graph theoretic model that is capable of capturing key biological information. We applied our model on five gut microbiome read data sets from patients with Crohn’s disease and eight gut microbiome data sets from healthy individuals. Focus produced excellent assembly results in an assembly comparison against the IDBA-UD, MetaVelvet, and Omega metagenomics assemblers. Graph mining revealed graph structural characteristics associated with biological features including rRNA operons and transposase sequences. A comparative study between the Crohn’s disease and healthy data sets revealed considerable differences in the phylogenetic distribution of conserved transposase sequences and associated antibiotics resistance genes. Previously the assembly graph has predominantly been used as a scaffold for the assembly process. In this study, we demonstrate that there is rich structural information contained within the overlap graph that can be extracted to make novel biological discoveries.

## Availability of data and materials

The data sets supporting the results found in this research article can be found in the SRA database under the accession numbers: SRR49544, SRR497943, SRR497952, SRR497643, SRR497648, SRR497650, SRR504939, SRR497646, SRR497657, SRR504939, SRR497946, SRR497948, SRR497949, SRR497645, SRR497652, SRR497654, SRR063543, SRR063544, SRR063545, SRR063587, SRR063588, SRR063589, SRR063903, SRR063904, SRR061730, SRR061731, SRR063539, SRR063548, SRR063549, SRR063553, SRR063554, SRR063555, SRR063583, SRR063584, and SRR063585.

## Additional files

Additional file 1: Taxonomic read assignments. This file is a spreadsheet that contains the raw read counts assigned to the genera present in Human Microbiome Reference Genomes. (XLSX 230 kb) Additional file 2: Proportion of nodes with Shannon’s index scores greater than one for sequence feature functional classes. This spreadsheet file contains the counts for sequence features assigned to various functional classes, including the Seed subsystems, RNA operons, and transposase sequences. This file also contains the proportion of sequence features whose nodes have Shannon’s index scores greater than one. The results of the Wilcoxon Signed-Rank test comparing proportions of nodes with Shannon’s index scores greater than one for the Seed subsystems against the transposase sequence features and RNA operons are also included in this file. (XLSX 74 kb) Additional file 3: Distribution of genera for the generated transposase clusters. This file is a spreadsheet with the distribution of the genera found in each generated transposase cluster for the Crohn’s disease and healthy data sets. (XLSX 57 kb) Additional file 4: Antibiotics resistance gene class enrichments. This spreadsheet file contains the counts for hits to antibiotics resistance gene classes in the generated transposase clusters. A Fisher’s test with FDR correction was used to determine if there were significant antibiotics resistance gene class enrichments for any of the clusters. (XLSX 51 kb) Additional file 5: Antibiotics resistance gene hits. This spreadsheet file contains the antibiotics resistance gene hits for each of the transposase clusters. (XLSX 55 kb) Additional file 6: Total genome length estimation for NG50. This spreadsheet file contains the calculation of the average genome lengths for the complete reference sequences available through the NCBI RefSeq database for the most abundant genera in the Crohn’s and healthy data sets. The estimated total genome length is also calculated in this file. (XLSX 21 kb)

## References

[1] O’Hara AM, Shanahan F (2006). The gut flora as a forgotten organ. EMBO Rep.

[2] Karlsson FH, Tremaroli V, Nookaew I, Bergström G, Behre CJ, Fagerberg B, Nielsen J, Bäckhed F. Gut metagenome in European women with normal, impaired and diabetic glucose control. Nature. 2013;498:99–103. 10.1038/nature12198 23719380

[3] Turnbaugh PJ, Ley RE, Mahowald MA, Magrini V, Mardis ER, Gordon JI (2006). An obesity-associated gut microbiome with increased capacity for energy harvest. Nature.

[4] Chung H, Pamp SJ, Hill JA, Surana NK, Edelman SM, Troy EB, Reading NC, Villablanca EJ, Wang S, Mora JR et al. Gut immune maturation depends on colonization with a host-specific microbiota. Cell. 2012;149:1578–93. 10.1016/j.cell.2012.04.037 PMC3442780 22726443

[5] Ivanov II, Littman DR (2011). Modulation of immune homeostasis by commensal bacteria. Curr Opin Microbiol.

[6] Schwabe RF, Jobin C (2013). The microbiome and cancer. Nat Rev Cancer.

[7] Ahn J, Sinha R, Pei Z, Dominianni C, Wu J, Shi J, Goedert JJ, Hayes RB, Yang L. Human gut microbiome and risk of colorectal cancer. J Natl Cancer Inst. 2013;105(24):1907–11. djt300. 10.1093/jnci/djt300 PMC3866154 24316595

[8] Illumina. [http://systems.illumina.com/systems/sequencing.html]

[9] 454 Sequencing. [http://454.com/products/index.asp]

[10] PacBio. [http://www.pacb.com]

[11] Paszkiewicz K, Studholme DJ (2010). De novo assembly of short sequence reads. Brief Bioinform.

[12] Peng Y, Leung HC, Yiu S-M, Chin FY. IDBA–a practical iterative de Bruijn graph de novo assembler. In Research in Computational Molecular Biology. Berlin Heidelberg: Springer; 2010. p. 426–40.

[13] Bankevich A, Nurk S, Antipov D, Gurevich AA, Dvorkin M, Kulikov AS (2012). SPAdes: a new genome assembly algorithm and its applications to single-cell sequencing. J Comput Biol.

[14] Simpson JT, Durbin R (2010). Efficient construction of an assembly string graph using the FM-index. Bioinformatics.

[15] Nagarajan N, Pop M (2013). Sequence assembly demystified. Nat Rev Genet.

[16] Myers EW (2005). The fragment assembly string graph. Bioinformatics.

[17] Haider B, Ahn T-H, Bushnell B, Chai J, Copeland A, Pan C (2014). Omega: an Overlap-graph de novo Assembler for Metagenomics. Bioinformatics.

[18] Peng Y, Leung HC, Yiu S-M, Chin FY (2012). IDBA-UD: a de novo assembler for single-cell and metagenomic sequencing data with highly uneven depth. Bioinformatics.

[19] Lai B, Ding R, Li Y, Duan L, Zhu H (2012). A de novo metagenomic assembly program for shotgun DNA reads. Bioinformatics.

[20] Afiahayati, Sato K, Sakakibara Y. etaVelvet-SL: an extension of the Velvet assembler to a de novo metagenomic assembler utilizing supervised learning. DNA Res. 2014: 22(1):69-77. 10.1093/dnares/dsu041 PMC4379979 25431440

[21] Namiki T, Hachiya T, Tanaka H, Sakakibara Y (2012). MetaVelvet: an extension of Velvet assembler to de novo metagenome assembly from short sequence reads. Nucleic Acids Res.

[22] Warnke J, Ali H. Focus: a new multilayer graph model for short read analysis and extraction of biologically relevant features. In Proceedings of the 5th ACM Conference on Bioinformatics, Computational Biology, and Health Informatics. New York, NY, USA: ACM; 2014. p. 489-98.

[23] Mahillon J, Chandler M (1998). Insertion sequences. Microbiol Mol Biol Rev.

[24] Klappenbach JA, Saxman PR, Cole JR, Schmidt TM (2001). rrndb: the Ribosomal RNA Operon Copy Number Database. Nucl Acids Res.

[25] Sommer MO, Dantas G, Church GM (2009). Functional characterization of the antibiotic resistance reservoir in the human microflora. Science.

[26] Sommer MO, Church GM, Dantas G (2010). The human microbiome harbors a diverse reservoir of antibiotic resistance genes. Virulence.

[27] Kurokawa K, Itoh T, Kuwahara T, Oshima K, Toh H, Toyoda A (2007). Comparative Metagenomics Revealed Commonly Enriched Gene Sets in Human Gut Microbiomes. DNA Res.

[28] Huddleston JR (2014). Horizontal gene transfer in the human gastrointestinal tract: Potential spread of antibiotic resistance genes. Infect Drug Resist.

[29] Ogura Y, Bonen DK, Inohara N, Nicolae DL, Chen FF, Ramos R (2001). A frameshift mutation in NOD2 associated with susceptibility to Crohn’s disease. Nature.

[30] Stecher B, Denzler R, Maier L, Bernet F, Sanders MJ, Pickard DJ (2012). Gut inflammation can boost horizontal gene transfer between pathogenic and commensal Enterobacteriaceae. Proc Natl Acad Sci.

[31] Bermejo F, Garrido E, Chaparro M, Gordillo J, Mañosa M, Algaba A, López-Sanromán A, Gisbert JP, García-Planella E, Guerra I et al. Efficacy of different therapeutic options for spontaneous abdominal abscesses in Crohn’s disease: are antibiotics enough? Inflamm Bowel Dis. 2012;18:1509–14. 10.1002/ibd.21865 22674826

[32] Costelloe C, Metcalfe C, Lovering A, Mant D, Hay AD (2010). Effect of antibiotic prescribing in primary care on antimicrobial resistance in individual patients: systematic review and meta-analysis. BMJ.

[33] Vigna S. Broadword implementation of rank/select queries. Experimental Algorithms [Internet]. Springer; 2008. p. 154–68. Available from: http://link.springer.com/chapter/10.1007/978-3-540-68552-4_12. [cited 2016 Mar 23]

[34] Larsson NJ, Sadakane K (2007). Faster suffix sorting. Theor Comput Sci.

[35] Warnke J, Ali HH. An efficient overlap graph coarsening approach for modeling short reads. Bioinformatics and Biomedicine Workshops (BIBMW), 2012 IEEE International Conference on: 4-7 October 2012. 2012. p. 704–11. doi: 10.1109/BIBMW.2012.6470223.

[36] Karypis G, Kumar V (1998). A fast and high quality multilevel scheme for partitioning irregular graphs. SIAM J Sci Comput.

[37] Zerbino DR, Birney E (2008). Velvet: algorithms for de novo short read assembly using de Bruijn graphs. Genome Res.

[38] Chao A, Shen T-J (2003). Nonparametric estimation of Shannon’s index of diversity when there are unseen species in sample. Environ Ecol Stat.

[39] Altschul SF, Madden TL, Schäffer AA, Zhang J, Zhang Z, Miller W, Lipman DJ. Gapped BLAST and PSI-BLAST: a new generation of protein database search programs. Nucleic Acids Res. 1997;25:3389–402. 10.1093/nar/25.17.3389 PMC146917 9254694

[40] Overbeek R, Begley T, Butler RM, Choudhuri JV, Chuang H-Y, Cohoon M, de Crécy-Lagard V, Diaz N, Disz T, Edwards R et al. The subsystems approach to genome annotation and its use in the project to annotate 1000 genomes. Nucleic Acids Res. 2005;33:5691–702. 10.1093/nar/gki866 PMC1251668 16214803

[41] Leplae R, Hebrant A, Wodak SJ, Toussaint A (2004). ACLAME: A CLAssification of Mobile genetic Elements. Nucleic Acids Res.

[42] Buchfink B, Xie C, Huson DH (2015). Fast and sensitive protein alignment using DIAMOND. Nat Methods.

[43] McArthur AG, Waglechner N, Nizam F, Yan A, Azad MA, Baylay AJ, Bhullar K, Canova MJ, Pascale GD, Ejim L, Kalan L, King AM, Koteva K, Morar M, Mulvey MR, O’Brien JS, Pawlowski AC, Piddock LJV, Spanogiannopoulos P, Sutherland AD, Tang I, Taylor PL, Thaker M, Wang W, Yan M, Yu T, Wright GD. The Comprehensive Antibiotic Resistance Database. Antimicrob Agents Chemother. 2013;57:3348–57. 10.1128/AAC.00419-13 PMC3697360 23650175

[44] Morgan XC, Tickle TL, Sokol H, Gevers D, Devaney KL, Ward DV, Reyes JA, Shah SA, LeLeiko N, Snapper SB et al. Dysfunction of the intestinal microbiome in inflammatory bowel disease and treatment. Genome Biol. 2012;13:R79. 10.1186/gb-2012-13-9-r79 PMC3506950 23013615

[45] NCBI SRA [http://www.ncbi.nlm.nih.gov/sra]

[46] Li H, Durbin R (2009). Fast and accurate short read alignment with Burrows–Wheeler transform. Bioinformatics.

[47] Consortium HMJRS (2010). A catalog of reference genomes from the human microbiome. Science.

[48] Nagalingam NA, Lynch SV (2012). Role of the microbiota in inflammatory bowel diseases. Inflamm Bowel Dis.

[49] Willing BP, Dicksved J, Halfvarson J, Andersson AF, Lucio M, Zheng Z, Järnerot G, Tysk C, Jansson JK, Engstrand L. A pyrosequencing study in twins shows that gastrointestinal microbial profiles vary with inflammatory bowel disease phenotypes. Gastroenterology. 2010;139:1844–54. 10.1053/j.gastro.2010.08.049 20816835

[50] Mondot S, Kang S, Furet J-P, Aguirre de Cárcer D, McSweeney C, Morrison M, Marteau P, Dore J, Leclerc M. Highlighting new phylogenetic specificities of Crohn’s disease microbiota. Inflamm Bowel Dis. 2011;17:185–92. 10.1002/ibd.21436 20722058

[51] Gevers D, Kugathasan S, Denson LA, Vázquez-Baeza Y, Van Treuren W, Ren B (2014). The Treatment-Naive Microbiome in New-Onset Crohn’s Disease. Cell Host & Microbe..

[52] Cho I, Blaser MJ (2012). The human microbiome: at the interface of health and disease. Nat Rev Genet.

[53] Rho M, Tang H, Ye Y (2010). FragGeneScan: predicting genes in short and error-prone reads. Nucleic Acids Res.

[54] Huang Y, Gilna P, Li W (2009). Identification of ribosomal RNA genes in metagenomic fragments. Bioinformatics.

[55] Nakano V, do Nascimento e Silva A, Merino VRC, Wexler HM, Avila-Campos MJ (2011). Antimicrobial resistance and prevalence of resistance genes in intestinal Bacteroidales strains. Clinics (Sao Paulo).

[56] Earl D, Bradnam K, John JS, Darling A, Lin D, Fass J (2011). Assemblathon 1: a competitive assessment of de novo short read assembly methods. Genome Res.

[57] Meyerson M, Gabriel S, Getz G (2010). Advances in understanding cancer genomes through second-generation sequencing. Nat Rev Genet.

